# Development of a Spectacle Wear Monitor System: SpecsOn Monitor

**DOI:** 10.1167/tvst.10.12.11

**Published:** 2021-10-06

**Authors:** Jayshree South, Paul Roberts, Tina Gao, Joanna Black, Andrew Collins

**Affiliations:** 1 School of Optometry and Vision Science, The University of Auckland, Auckland, New Zealand; 2 Medlink Innovation Limited, Auckland, New Zealand, Auckland Bioengineering Institute, The University of Auckland, Auckland, New Zealand

**Keywords:** compliance, amblyopia, spectacle-wear, monitoring

## Abstract

**Purpose:**

This study aimed to custom design, build, and test a removable device that accurately and objectively monitors adherence to spectacle wear in preschool children participating in clinical trials. This work will provide researchers with the tools to investigate the effect of adherence to optical treatment in conditions relating to refractive error, such as anisometropia, amblyopia, myopia, and accommodative esotropia, where spectacle wearing behaviors are of interest.

**Methods:**

Several sensors were considered in the design of the SpecsOn monitor. The final version included two temperature sensors, one that measures skin temperature through an infrared sensor directed at the wearer's temple on the spectacle arm and the other measuring device temperature. The difference between the two temperature readings is used to determine if the spectacles were worn. The SpecsOn monitor was tested in two phases in adult participants (laboratory *n* = 10 and real world *n* = 5).

**Results:**

Results from both phases showed good agreement between the objective measurement of wear based on skin and device temperature differences and participants’ manually logged wear times. The custom built SpecsOn monitor was 99% successful in accurately detecting spectacle wear in our adult cohort.

**Conclusions:**

The SpecsOn monitor offers a convenient, accurate, and reliable system to monitor spectacle adherence. The devices were comfortable, secure, and unobtrusive to wear, and fitted easily to a variety of frame styles.

**Translational Relevance:**

Easy access to spectacle compliance information from the SpecsOn monitor during the optical treatment phase will optimize visual outcomes and provide detailed clinical data to support decision making on the need and timing of additional therapies, improving treatment efficiency.

## Introduction

A common challenge when prescribing spectacles for children in conditions such as amblyopia (decreased acuity in the absence of pathology) is poor spectacle adherence (compliance) to prescribed wear time. Approximately 69% to 80% of children with amblyopia have refractive error in at least one eye.^1^^–^^4^ Adherence to full-time spectacle wear is essential for optimal outcomes from the optical treatment phase and can affect the commencement of additional and adjunct treatment.^5^^–^^8^

In current clinical settings, adherence with spectacle wear is only assessed indirectly and subjectively via parental reporting,^9^^,^^10^ which is generally expected to overestimate adherence. Medical nonadherence, also found in other aspects of prescribed medical interventions, imposes a considerable financial burden on health care systems.^11^^,^^12^ Current amblyopia research shows a wide variability in adherence to occlusion therapy.^13^^–^^16^ Adherence with spectacle wear also displays a similar range of interindividual variability with a potential dose–response relationship with visual improvements directly correlated with hours of spectacle wear.^17^ If first-line optical treatment is made more effective, then this strategy would decrease the number of children needing patching and atropine treatments, shortening treatment time, decreasing treatment burden, and be a substantial cost saving to health systems.

A review of the literature reveals few existing objective spectacle adherence monitors. Monitors described were often modified from their original purpose, such as thermal sensors designed for monitoring transportation temperatures of foods or laboratory materials,^18^ detecting wear of orthodontic appliances,^19^ and monitoring the wear of eye patches (occlusion dose monitors).^10^^,^^17^^,^^20^ Early iterations of research-purposed sensors were large bulky devices that were not aesthetically pleasing,^10^^,^^20^ which was shown to negatively impact adherence in children.^21^ Battery life and data storage were also limited and data evaluation described as “laborious.”^22^ Newer sensors like the SmartButton data logger^17^ and the TheraMon orthodontic microsensor^18^ are two systems that have been repurposed to monitor adherence with spectacle wear^18^^,^^19^; these devices are smaller and lighter than previous devices. These sensors take continuous temperature measurements, where a significant change in temperature is used to determine whether the spectacles are on or off. These devices have, however, only been tested in an adult population.^18^^,^^19^ The positioning of the sensors at the end of the spectacle arm behind the ear makes the monitors more discreet. However, in a preschool population where amblyopia treatment is often initiated, they are a potential swallowing or choking hazard given that children are prone to chew on the end of spectacle arm where the sensor is placed. Temperature sensors are known to have a higher rate of false-positive readings if held in the hand, in a pocket, or placed in a warm environment,^22^ such as a car parked outside.^18^ Sensors like the SmartButton and TheraMon, which measure a single temperature, are also more susceptible to error-inducing manipulations and false readings.

Liquid crystal, “shutter glasses,” offer an alternate approach to amblyopia treatment. A new electronic frame designed to be used with the shutter glasses contains a combination of temperature and capacitive sensors and can detect wear time, occlusion time, and measure the state of wear position.^23^ However, to date this remains a proof-of-concept design. With recent technological advancements in myopia research, a number of wearable devices have been developed to provide real-time objective measures of light intensity, physical activities, and distance to reading material through use of light, gyroscopic, acceleration, and infrared sensors.^24^^,^^25^ These sophisticated multifunctional devices are designed to fit a variety of frames, but again are not designed to specifically measure spectacle adherence. The devices require regular recharging, posing a burden on parents to remember to recharge the device, which risks lost data. In addition, the devices are not designed from a safety perspective for a younger population. These devices are also expensive.

The aim of this study was to custom design and pilot test a removable device (integrating sensors, microprocessor, data storage, and energy source) that accurately and objectively monitors spectacle wear in an adult cohort to provide validation before use in a preschool population. Although spectacle wear behavior is not different between adults and children, the pilot study recruited adult participants to prove the design concept and to ensure data recorded could reliably be compared with diary entries. The SpecsOn device can also be applied to a wide range of other clinical and research applications in the treatment of childhood vision conditions that require spectacle treatment such as myopia, hyperopia, and accommodative esotropia.

## Methods

### System Design

We required an accurate monitoring system that was compact, would attach securely to most frames, and was safe, following medical device safety guidelines for children (Medsafe NZ and European standards^26^^–^^30^ guidelines). Data collected needed to be stored on-device for at least 6 weeks, the typical clinical follow-up period for amblyopia therapies, so an adequate power supply was also required.

**Figure 1.**
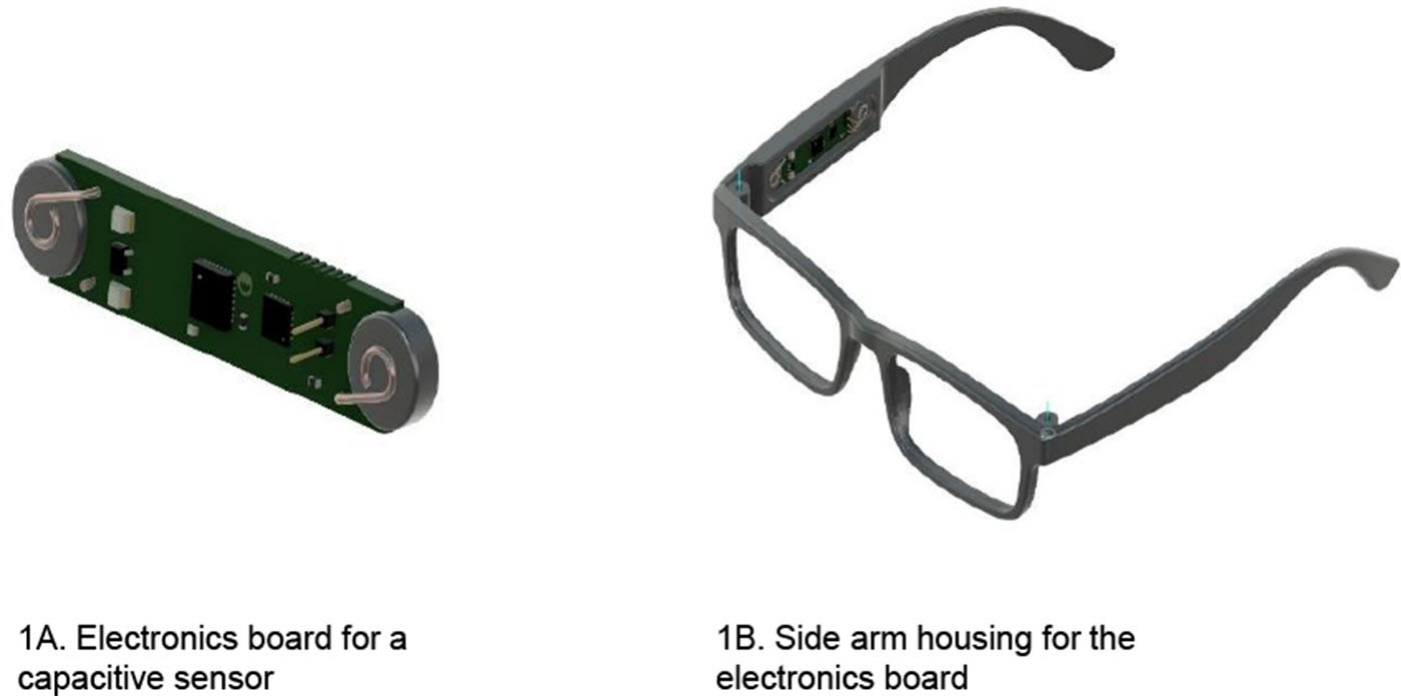
Computer-aided designs for a capacitive sensor system. (A) Electronics board for a capacitive sensor. (B) Side arm housing for the electronics board.

During the design phase, we considered several types of sensors such as mechanical, proximity, magnetometer, accelerometers, and other biosensors such as pulse oxygen monitors. However, devices these were either susceptible to false-positive readings, require intensive computation to determine motion, or were difficult to implement physically. For greater accuracy we initially created a prototype using a touch-sensitive capacitive sensor (similar to smartphone screens) embedded in the nose pad of the spectacle frame. The nose pad position was chosen because it would likely generate the least number of false positives compared with placing elsewhere on the frame. The electronic components were to be discreetly housed internally in a three-dimensional printed side arm of the spectacle frame (Fig. 1). However, after initial testing of the capacitive sensor prototype, several factors made us rethink this approach. The capacitive sensor in the nose pad design required the frame to be a part of the circuitry connecting the sensor to the processer. This factor meant the device could only be used with metal frames or if conductive material was incorporated into plastic frames. This did not fulfil our requirement for the device to be easily adaptable to a wide variety of frames, because the design required side arms to be three-dimensional printed individually.

**Figure 2.**
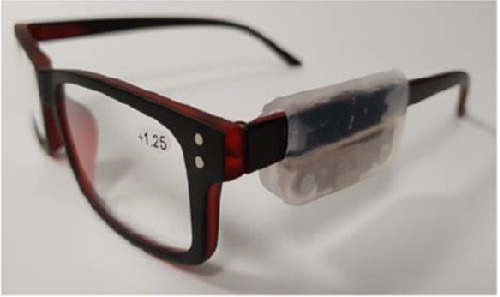
SpecsOn monitor attached to the side arm of the spectacle frame.

The final version, the SpecsOn monitor, is externally mounted under the arm of the spectacles (Fig. 2) using a detector incorporating two temperature sensors with 0.02 °C resolution and 0.5 °C accuracy (MLX90615, Melexis NV, Leper, Belgium). One sensor, directed at the wearer's temple measures skin temperature using an infrared detector, the other measuring the device's temperature as an estimate of the ambient temperature. Temperature measurements are taken at 1-minute intervals for phase one testing and 5-minute intervals for phase two testing and written to nonvolatile memory with 12 weeks of storage capacity. The device is battery powered with a capacity for 15 weeks recording. Analysis of the two temperature measures can determine if the spectacles are being worn. These components are safely enclosed in a water resistant skin-safe silicone casing, which allows the monitor to fit a majority of spectacle frames. Data are downloaded from memory via physical connector and a USB interface unit and interpreted via custom analysis software.

### Participants

This study was approved by the University of Auckland Human Participants Ethics Committee (Reference number 023301) and adhered to the principles of the Declaration of Helsinki. Adult participants for both phases were recruited from the students and staff at the University of Auckland School of Optometry and Vision Science. All participants in both phases were self-reported to be free of eye disease and written informed consent was obtained for all participants.

### Phase One: Laboratory Based

Adult participants who wore contact lenses or were non or part-time spectacle wears were asked to sit and watch a short movie or continue with their normal computer-based tasks for 60 minutes (in the laboratory or an office setting) while wearing a pair of study frames with the SpecsOn monitor attached. In this phase, temperature measurements were taken at 1-minute intervals. A researcher remained in the room and asked the participant to put on or remove the study spectacles according to a predetermined schedule and this process was manually recorded by the researcher.

### Phase Two: Real World

Adult participants who habitually wore spectacles full time or part time were recruited. The SpecsOn device was attached to the side arm of their spectacles (see Fig. 2), and they were asked to wear their spectacles as usual under normal conditions. For one participant, this practice happened to include when they travelled to Fiji. The SpecsOn device was adjusted to measure temperature at 5-minute intervals to optimize data storage. Participants were provided with a diary and asked to record when they wore and removed their spectacles over a 7-day period. Participants were also asked to record a general description of activities and environmental conditions each day and where spectacles were stored when not worn, to compare the effects of different activities such as exercise, weather conditions such as wind or rain, and any relevant environmental factors like ambient temperature.

### Data Analysis

Data were downloaded from the device's memory via a USB interface and saved to a computer using custom software created with LabVIEW 2013 (National Instruments, Austin, TX). Wear times were identified from the temperature differential between the skin and the device sensors and compared with the manual log kept by participants. To confirm whether the SpecsOn monitor had accurately captured the spectacle-wear in phase one, a custom program (MATLAB R2018b, MathWorks Inc, Natick, MA) was used to calculate wear time and compare against the researcher's manual records of when the spectacles were put on or removed. Spectacles were considered on if the skin temperature was 4 °C greater than the device temperature. Data from the temperature sensors were distributed normally.

**Figure 3.**
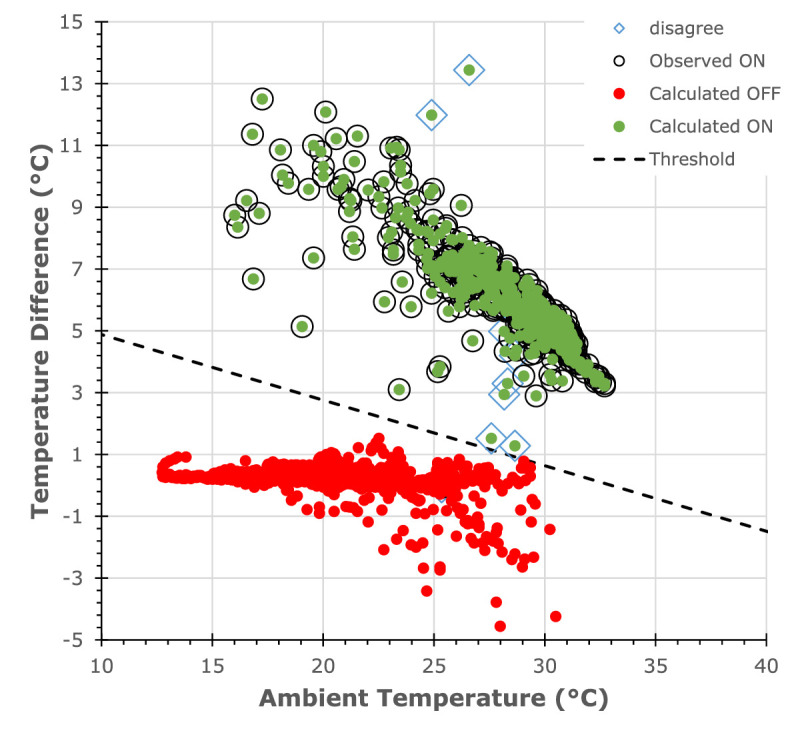
Sample of the threshold analysis plot from participant JS1 in phase 2. Shows good agreement, between the manual logs (observed ON) and the calculated wear time (calculated ON).

For the analysis of phase two data, the calculation method was changed to threshold technique to improve correlation between actual and calculated wear times and to account for changes in ambient temperature during day to day activities. The threshold, Equation 1, was determined after plotting the temperature differential against ambient temperature for all participants. An example of this data is shown in Figure 3. Data above the threshold represented intervals when spectacles were identified as being worn, those below represent the spectacles were off. Microsoft Excel was used to perform this analysis. and compare data from manual logs to verify correct wear times for each participant.

(1) $$\;\;\;\begin{array}{c} \Delta t>\left(\left(-0.21\times Ambient\;Temperature\right)+7\right)=\mathrm{Spectacles}\;\mathrm{On} \end{array}$$

## Results

All participants in phase one (*n* = 10) and in phase two (*n* = 5) completed data collection. The results from both phases show good agreement between the threshold temperature differential method and the manual logged wear times.

### Phase One Results

The mean wear time temperatures from the skin sensor was 33.6 ± 0.75 °C and from the device sensor was 26.3 ± 1.05 °C for phase one testing. The mean temperature differential between the two sensors during wear was 7.2 ± 1.59 °C across all 10 participants. Wear time was calculated based on a temperature differential of greater than 4 °C difference between the skin and the device temperature (Fig. 3). Good agreement between the manual logs and the calculated wear time shows the SpecsOn device was accurately measuring spectacle wear time.

**Figure 4.**
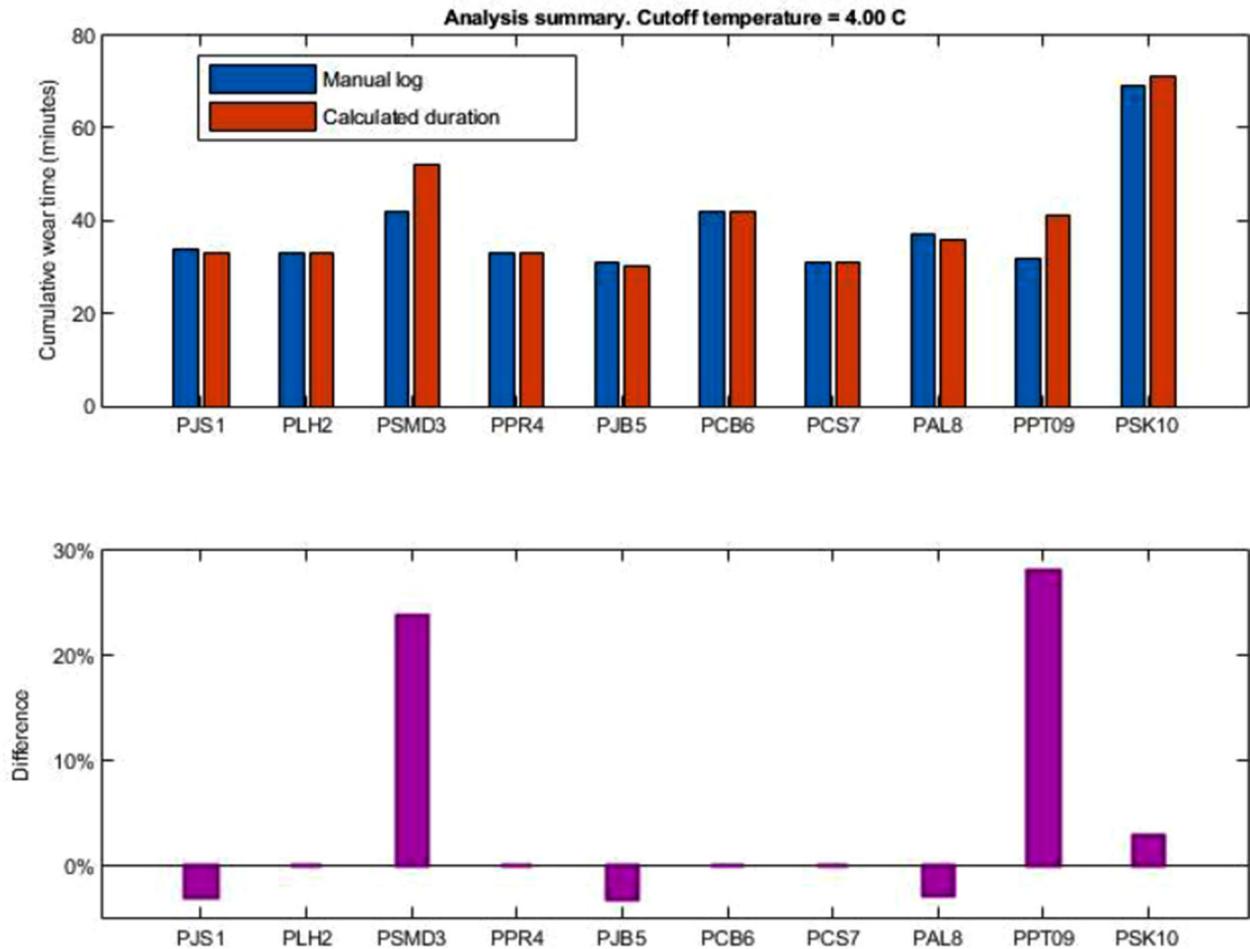
Good agreement using a 4 °C differential between skin and device temperature and the manually recorded log to calculate wear time.

**Figure 5.**
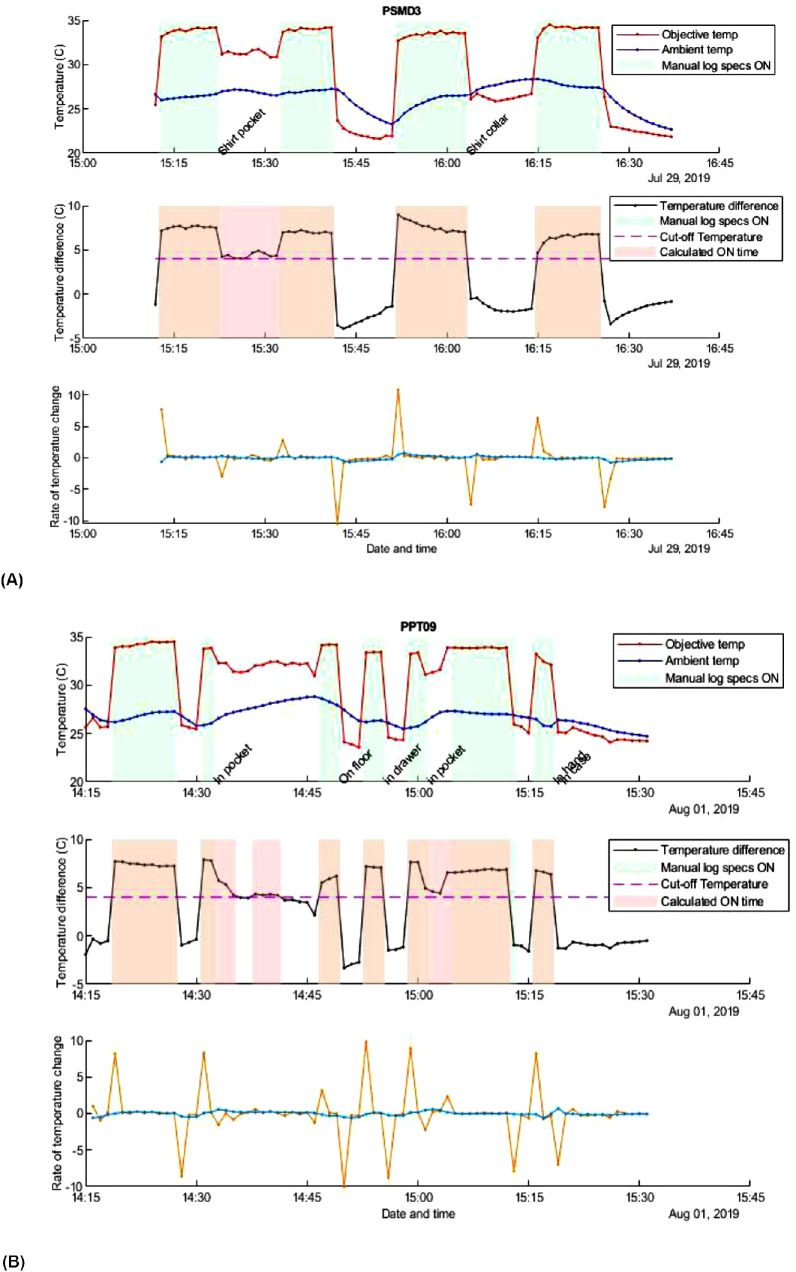
False-positive error for participants (A) PSMD3 and (B) PPT09 calculated wear time from spectacles being placed in shirt pockets.

Two participants (PSMD3 and PPT09) had a higher percentage difference between the calculated wear time and the manually recorded logs (Fig. 4). These participants were asked to place the spectacles in shirt pockets during phase one testing to see if this common behavior would generate a false-positive error. The temperature differential decreased slightly when the spectacles were placed in the pockets and it was more difficult to determine a calculated wear time based on the 4 °C differential (Fig. 4). However, on closer examination of the data, it was clear to see where the temperature of the skin sensor decreased slightly as the spectacles were removed and placed into the pocket. The time the spectacles were kept in the pocket also recorded a lower skin temperature that was distinguishable from when they were being worn on the face (Fig. 5).

### Phase Two Results

During phase two, the devices were subject to a wider range of environmental conditions and routine spectacle wear behaviors to determine further potential sources of errors. Extreme activities such as placing the spectacles close to a fireplace for 30 minutes where the skin temp ranged from 25.9 °C to 45.0 °C and the device temp ranged 24.6 °C to 26.6 °C or putting the spectacles in the fridge for 20 minutes where the skin temp range was 6.1 °C to 10.3 °C and the device temp range was 8.9 °C to 20.6 °C did not cause a false-positive error. Other common spectacle wear behaviors, such as placing glasses in a shirt, jacket, or trouser pocket, being hung off a shirt, collar and spectacles folded and held in the hand (with a hand near the sensor) did not cause false-positive errors. Placing spectacles on the forehead or on top of the head did result in “Calculated ON” errors, which are shown as the “Disagree” points in Figure 3. However, looking closely at the raw data (Table 1) a significant decrease in the temperature differential from 7.8 °C to 2.9 °C is evident and this lower differential was maintained while the spectacles were placed on top of the head (2.9 °C, 3.3 °C, and 4.2 °C) before rapidly decreasing once the spectacles were removed. Even in warmer climates (Fiji, 18 °C–37 °C as opposed to New Zealand, 12 °C–35 °C) the device was able to accurately detect spectacle wear (Supplementary Material), but wear time was difficult to calculate based on the 4 °C temperature differential cutoff. Therefore, a threshold analysis, as described elsewhere in this article, was used to improve accuracy for in warmer ambient temperatures. Overall, there was 99% agreement between the calculated wear time based on threshold temperature differential and the detailed manual logs for all five participants (Table 2).

**Table 1.** Sample Log Showing a Decrease in the Differential Temperature When the Spectacles Were Moved to be Placed on Top of the Head

Skin Temperature	Device Temperature	Temperature Differential	Diary On/Off	Observed On/Off	Calculated On/Off
31.85	22.51	9.34	On	On	On
34.35	26.59	7.76	On	On	On
31.11	28.17	2.94	Off on head	Off	On
31.63	28.33	3.3	Off on head	Off	On
32.85	28.65	4.2	Off on head	Off	On
23.43	27.99	−4.56	Took off	Off	Off
22.63	24.49	−1.86	Off	Off	Off
22.53	22.95	−0.42	Off	Off	Off

**Table 2.** Success Rate for Detecting Overall Spectacle Wear Using the SpecsOn Monitor

Participants	Calc ON (h)	Calc OFF (h)	Calc total h (Calc ON + Calc OFF)	Logged Total (h)	Disagree (h)	Agreement Between Calculated and Logged (%)
PR	51.5	357.7	409.2	409.2	2.0	99.51
JSNZ	39.3	393.3	432.6	439.2	2.2	99.50
AC	54.7	97.6	152.3	167.0	1.0	99.34
TG	80.3	107.8	188.0	187.9	0.3	99.82
JB	87.2	122.6	209.8	209.2	1.1	99.48
JSFJ^*^	2.8	217.8	220.6	221.8	1.3	99.40

* Data from Fiji for participant JSFJ.

## Discussion

Strong agreement between reported wear time and calculated wear time determined by a temperature differential threshold shows the SpecsOn monitor is 99% accurate in monitoring spectacles wear in an adult cohort during a variety of routine activities and ambient temperatures. The device was comfortable, secure, and unobtrusive to wear, and easily fitted to a variety of frame styles. Data were easily downloaded via a USB interface unit and interpreted into clinically useful data, such as total wear time.

The final version of the SpecsOn monitor included two temperature sensors, one located internally within the silicone packaging to estimate the ambient temperature and an externally facing infrared temperature sensor to measure skin temperature of the wearer's temple. This version was revised from our original concept based on capacitive sensing, which was initially considered to be advantageous over temperature sensing because capacitive would only detect wear when in direct contact with skin. However, the high noise susceptibility of the small capacitive sensors, complexities integrating suitable sensor pads, and the cost of producing an effective system (including three-dimensional printed side arms to house components for a variety of frames) made us rethink our approach.

Skin contact temperature sensors have previously been used in studies to measure spectacle wear with varying success.^17^^–^^19^ The SmartButton temperature datalogger is one such device which was found to have an 80% success rate in detecting overall spectacle wear in adults.^18^ However, without a matching participant log, it was difficult to confirm wear time patterns. Sensor loss owing to double-sided adhesive failure and skin irritation from the silicone mounting against the skin were the main reasons for failure to collect data in that study. The Glasses Dose Monitor is another system that had been adapted from the coin sized occlusion dose monitor first described by Simonsz et al.^20^ It is composed of two thermistors that measure the temperature difference between the front and the back of the sensor. A very small difference threshold of 0.3 °C was used to indicate when the spectacles were being worn. Only 83% of the monitors in that study were successful in collecting data and, again, the main cause of failure was detachment and loss of the monitor. This nature presents a potential health and safety hazard because small detachable parts pose an ingestion or inhalation hazard^31^ to the preschool population for whom amblyopia treatment is commenced routinely. With 99% detection, the SpecsOn monitor is a considerably more accurate system for detecting overall spectacle wear. The skin safe silicone casing of the SpecsOn monitor securely adheres the monitor to the arm of the spectacle frames, making it significantly safer for use with the intended pediatric population.

Single temperature sensors used in previous studies have resulted in high false-positive rates when the ambient temperature has exceeded 33 °C^19^ or 37 °C.^18^ The advantage of the infrared sensor used in this study is that it only measures the temperature of the object that it is directed at (skin), a second sensor is included which accounts for the effects of ambient temperature changes. Even in a warmer ambient environment (Fiji), the SpecsOn monitor was able to detect wear using the temperature differential between the skin and the device temperature sensors and appropriate threshold analysis (see the Supplementary Material). The SpecsOn monitor was tested in ambient temperatures from 12 °C to 37 °C. It is possible for false-negative results to arise if the ambient temperature becomes hot enough to reach skin temperature, but this has not occurred in the temperate climate where this study was conducted.

The context-sensitive smart spectacles^23^ device is an electronic spectacle frame incorporating a combination of temperature and capacitive sensors. These sensors detect the position of liquid crystal shutter glasses, used as an alternative to amblyopia treatment. They also detect when glasses were removed and incorporate recognition of activities such as walking, sitting, jumping, and so on. A small pilot study of this sophisticated design found a 91.4% agreement in detecting the correct position of glasses when worn and a 100% agreement in detecting when the glasses were taken off. The main aim of these spectacles is to monitor adherence to an alternative method of occlusion therapy in amblyopia. It could be adapted to monitoring adherence during the refractive adaption phase; however, the production of these electronic frames are still in the concept phase and likely to be expensive and difficult to accommodate different spectacle frames. Two very recent wearable objective measuring devices have become available. The Clouclip^32^^,^^33^ objectively measures near-work distance and duration in the investigation and prevention of myopia progression. It provides vibration alerts if the near-work activity is too close or if the duration of near work exceeds acceptable time limits. It incorporates a triaxial accelerometer that differentiates between wear and not wear states. However, the device cannot measure duration of wear alone and the accelerometer goes into sleep mode after 40 seconds if no change is detected. Therefore, it would be difficult to know if spectacles were being worn if a child were to be lying down and watching television or sitting still while engrossed in an activity. The Clouclip is a rechargeable device and is not too dissimilar in size to the SpecsOn monitor. However, the total battery life and time required to fully charge the device is not stated in the reporting literature.^32^^,^^33^ Having a rechargeable battery places a burden on parents to remember to recharge the device. An exposed recharge port also means the device is not water resistant, which would not be ideal for a preschool population. The Vivior^25^ is another recently developed device for measuring visual behavior in adult patients undergoing cataract and refractive lens exchange surgery to improve treatment outcomes. This device, however, is designed to be used short term and only has a recording capacity of 16 hours, which would not be ideal for amblyopia treatment monitoring. The device requires regular recharging and is also heavier, at 14 g, than the Clouclip and the SpecsOn devices, which could affect comfort and the positioning of a child's frame. The advantage of the SpecsOn device is that it is powered by primary batteries for a duration of 15 weeks. Even though routine follow-up visits during amblyopia therapy typically occur at 6-week intervals, visits may be delayed or missed, risking lost data. A 15-week battery life allows the monitoring device to capture data for the full duration of the optical treatment phase, at least 12 weeks. Having the SpecsOn device will not necessarily change the review interval because the review time is based on the expected progression of visual acuity during optical treatment. The SpecsOn monitor is specifically designed for monitoring spectacle adherence. It is relatively compact in comparison with existing available options, more adaptable to a variety of frames and easier to produce. We are currently using the SpecsOn monitor in a clinical trial for preschool aged children (MAGNIFY study ACTRN12620000061932). We hypothesize that temperature monitoring will accurately measure spectacle adherence in preschool-aged children.

The current design uses a USB interface to download the data, but there is potential to use a Bluetooth (wireless) connection and a mobile device app in the future. The clinically relevant data from the SpecsOn monitor are easily retrieved and analyzed. The data can be explored to review wear patterns such as overall adherence rate during waking hours, average weekly and daily wear times, through to the portion of each hour during a day that the spectacles were worn. This analysis allows accurate wear patterns to be determined and answer such questions as to whether the participants only wore their spectacles at school.

One of the limitations of this study is that the SpecsOn monitor is larger than conceptualized and, although it is small and discreet on an adult size frame, it may be more obvious on a child size frame. The final dimensions of the device were mainly due to limitations in decreasing the size of the hardware and components while maintaining sufficient battery capacity. We chose to use primary batteries instead of rechargeable, because we felt that expecting participants and parents to be responsible for recharging would be an extra burden, and risk missing recording data owing to flat batteries. A further size consideration was ensuring, in the unlikely event the participant removed the device from the spectacle frame, that the device's overall size complied with choke hazard standards for toys.^31^ The SpecsOn monitor casing is made of medical grade silicone and is designed to sit far enough forward on the side arm, close to the hinge, to prevent it from touching the side of the face and causing irritation. The positioning and the size of the monitor may make it cosmetically unappealing to some. To overcome this factor, we plan to color the silicone casing and allow participants to choose a color to match the frame, making it more discreet. There is also an option to emboss patterns on to the silicone casing to make it more child friendly.

## Conclusions

Spectacle adherence is correlated with visual improvements during optical treatment of amblyopia. The SpecsOn monitor offers a convenient, accurate, and reliable system that does not require recharging to monitor spectacle adherence in children for the full duration of the optical treatment phase. This strategy provides researchers with the tools to investigate factors influencing optical treatment such as adherence, wear patterns, and duration the refractive correction has been worn, which could influence treatment outcomes and provide information in relation to timings of adjunct therapies. There is also a wide range of other clinical applications possible for this system in the treatment of childhood vision conditions that require spectacle treatment, such as accommodative esotropia, hyperopia, or myopia.

## Supplementary Material

Supplement 1
