# Supplementary material for: Development of a Spectacle Wear Monitor System: SpecsOn Monitor
Source: Transl Vis Sci Technol. 2021 Oct 6;10(12):11. doi: 10.1167/tvst.10.12.11 (PMC8496409; doi:10.1167/tvst.10.12.11)

Supplementary Material

Sample of the threshold analysis plot of wear time in a warmer ambient temperature. Plot shows good agreement in detecting spectacle wear based on threshold analysis.

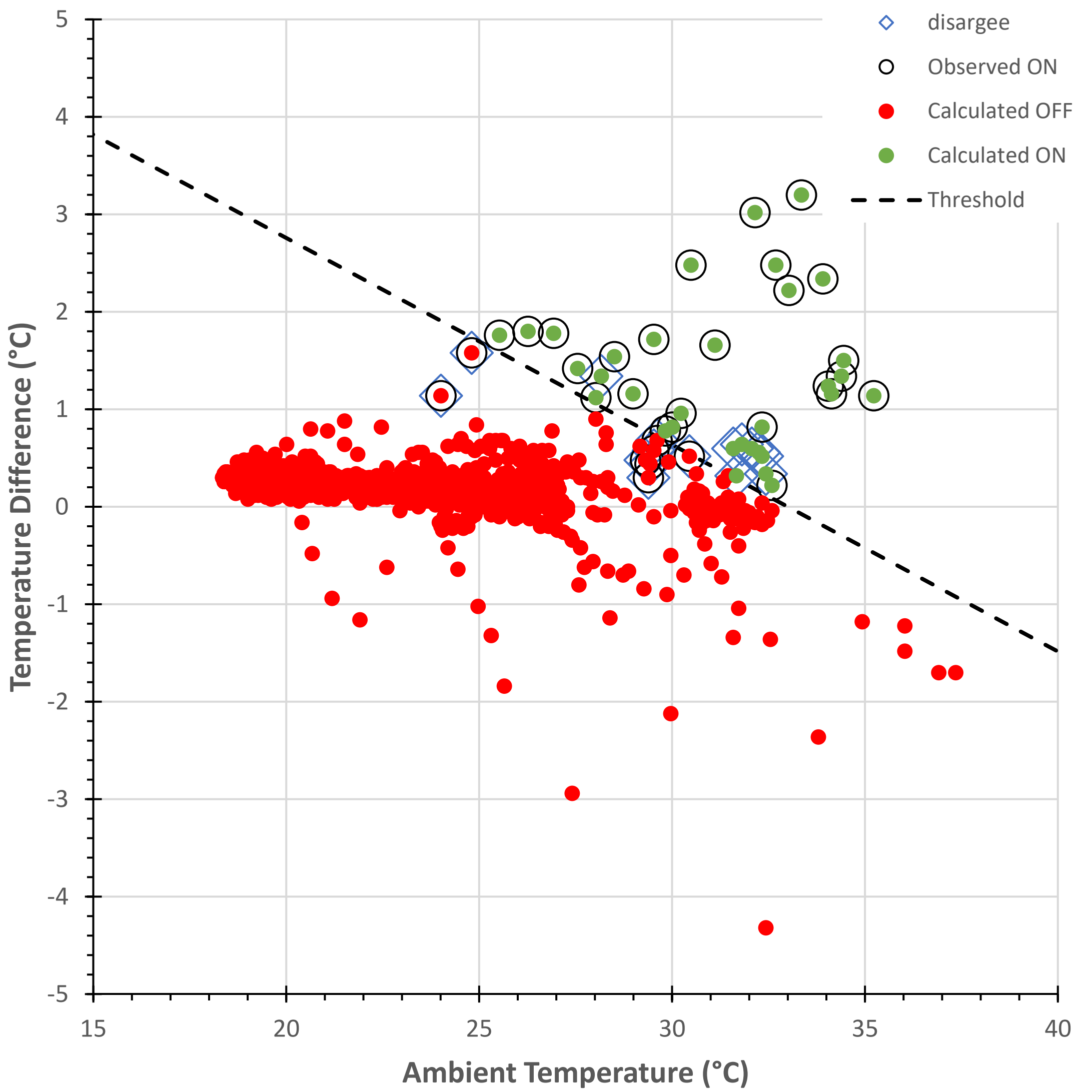

Supplement: Supplement 1 [file tvst-10-12-11_s001.pdf]
